# Bovine Natural Antibody Relationships to Specific Antibodies and *Fasciola hepatica* Burdens after Experimental Infection and Vaccination with Glutathione *S*-Transferase

**DOI:** 10.3390/vetsci9020058

**Published:** 2022-01-31

**Authors:** Gemma Zerna, Timothy C. Cameron, Hayley Toet, Terry W. Spithill, Travis Beddoe

**Affiliations:** Department of Animal, Plant and Soil Sciences and Centre for AgriBioscience, La Trobe University, Bundoora 3083, Australia; gmzerna@students.latrobe.edu.au (G.Z.); Tim.Cameron@latrobe.edu.au (T.C.C.); H.Toet@latrobe.edu.au (H.T.); T.Spithill@latrobe.edu.au (T.W.S.)

**Keywords:** natural antibodies, specific antibodies, bovine, KLH, glutathione *S*-transferase, *Fasciola hepatica*, helminth infection

## Abstract

*Fasciola hepatica* is the causative agent of fasciolosis, a significant parasitic disease occurring worldwide. Despite ongoing efforts, there is still no vaccine to control liver fluke infections in livestock. Recently, it has been suggested that natural antibodies (NAbs) can amplify specific antibodies (SpAb) and have a direct killing effect, but it is unknown if this phenomenon occurs during parasitic helminth infection or targeted vaccination. NAbs are antibodies produced by the innate immune system, capable of binding antigens without prior exposure. This study explores the role of bovine NAbs, using the exogenous glycoprotein keyhole limpet hemocyanin (KLH), in response to *F. hepatica* infection and SpAb production after infection and vaccination. The cattle’s NAbs were differently influenced by parasite infection and vaccination, with an increase in KLH-binding IgG and IgM levels after infection and reduced KLH-binding IgM levels following vaccination. Underlying NAbs reacting to KLH showed no correlations to the final fluke burdens after experimental infection or vaccination. However, NAbs reacting to whole-worm extract (WWE) prior to infection were positively correlated to increased fluke burdens within the infected bovine host. Furthermore, after infection, the specific IgG reacting to WWE was positively reflected by the underlying NAb IgG response. Following subcutaneous vaccination with *F. hepatica* native glutathione *S*-transferase (GST), there was a non-significant 33% reduction in fluke burden. Vaccinated animals with higher underlying NAbs had a higher induction of vaccine-induced SpAbs, with trends observed between KLH-binding IgM and anti-GST IgG and IgM. Our findings provide a platform to allow further investigation to determine if NAb levels could mirror fluke-SpAb production for exploitation in a combined selective breeding and vaccination program. Additionally, this work suggests that liver fluke could possibly evade the host’s immune system by utilising surface-bound IgM NAbs.

## 1. Introduction

The livestock industry is a vital global commodity under increasing pressure due to the growing population and food demands [1]. Helminth infections are a threat to efficient production and animal welfare [2,3]. The globally distributed *Fasciola* spp. cause upwards of US $3.2 billion per annum in lost revenue in livestock production and associated control measures [4,5]. Current control of liver fluke infections heavily relies on anthelmintics, particularly triclabendazole, but drug resistance to triclabendazole has now been reported on multiple continents [6,7]. These reports highlight the need to develop a sustainable and effective vaccine to diminish the production losses resulting from fasciolosis. Thus far, the majority of vaccine studies against *F. hepatica* resulted in a lack of significant efficacy and the repeatability required for commercialisation.

Glutathione *S*-transferases (GST) are enzymes found in almost all animals and act as immune-evasion molecules in helminths, as they are involved in cellular detoxification of drugs, protection against immune-induced damage and excretion of many xenobiotic substances [8]. GST could be a protein family involved in evading antibody dependent cellular cytotoxicity (ADCC), the fluke killing mechanism demonstrated in vitro [9,10]. GSTs reduce the effects of free radicals released by degranulating cells on juvenile flukes, by detoxifying the secondary products of lipid peroxidation that is produced from the host immune-initiated free-radical attack on the parasite [8,11,12,13]. Therapeutic neutralisation of parasite-secreted GSTs could therefore make the juvenile flukes more susceptible to cytotoxic attack [14]. Vaccination with native *F. hepatica* GST in Quil A and squalene Montanide® 80 (Seppic S.A., Paris, France) has shown partial protection in cattle, with a mean efficacy of 43% (range of 19–69%) in 8 vaccine trials [14]. Such variation in vaccine efficacy between trials has been seen in numerous *F. hepatica* vaccine trials testing other antigens but the reasons for this is poorly understood [12,15].

In recent years, there has been exploration into the role natural antibodies (NAbs) may play in influencing the production of pathogen-specific antibodies (SpAb), along with how NAb levels may influence the health status of animals. NAbs are described as antibodies capable of binding to antigens without a known prior exposure to the antigen [16]. NAbs are predominantly of the IgM isotype, with IgG and IgA also contributing to the repertoire, but to a lesser extent [17]. They have a low binding affinity but a broad specificity, typically towards conserved structures such as carbohydrates and phospholipids [18]. Underlying NAbs are proposed to be the first line of defence against pathogens, with abilities to interact and predominantly clear viral and bacterial infections (reviewed by [19,20]). The cellular production of NAbs is different from SpAbs, as NAbs are produced by the foetal and neonatal developed B1 B-cell subset that are long-lived, self-renewing and mainly localized within the peritoneal and pleural cavities [21,22]. In contrast, SpAbs are produced by B2 B-cells which are stimulated after exposure to a foreign antigen [23]. This makes NAbs part of the innate immune system and key to protection against invading pathogens from the early onset of infection while the adaptive immune system is establishing [16].

Prediction of livestock susceptibility to disease is important for the maintenance of animals and retaining productivity. NAbs are inheritable and selective breeding for high NAb levels can improve the general health status of chickens, with high-NAb-titre lines showing a 50–60% increased survival rate after challenge with avian pathogenic *E. coli* compared to the low-NAb line following selective breeding over four generations [24,25]. Furthermore, high-NAb chicken lines can show increased production of SpAbs after vaccination; however, this is dependent on the antigen generating SpAbs [26]. Bovine NAbs are inheritable and found to improve mastitis resistance, tolerance to disease and energy balances in lactating dairy cows [27,28,29,30,31]. Pools of sera from high-NAb-titre cattle have improved binding abilities to common microbial structures and lower rates of clinical mastitis than pools consisting of low-NAb responders [27,28]. Therefore, NAb levels can act as a potential marker for the health status of individual animals, are inheritable and can be used in breeding programs to improve disease resistance.

Due to the polyreactive nature of NAbs, they have been previously mistaken and dismissed as background binding in assays [32]. However, they are now being characterised through indirect enzyme-linked immunosorbent assay (ELISA), predominantly by their reactivity towards a high-molecular-mass glycoprotein, keyhole limpet hemocyanin (KLH) that is derived from the sea mollusc *Megathura crenulata*; since this mollusc is not found within livestock environments, cattle are unlikely to have prior natural exposure toward KLH [33].

As of yet, no studies have investigated the role of NAbs during helminth parasite infection in cattle. It would be invaluable to experimentally determine a naïve or underlying subset of antibodies that reflects the SpAb response following vaccination or parasite infection. This study assessed (A) the bovine NAb and SpAb (IgM and IgG) responses over the course of a GST vaccination trial; and (B) the NAb and SpAb (IgM and IgG) responses before and after experimental infection with *F. hepatica*. The aims were to determine if the underlying NAb repertoire can predict SpAbs stimulated after vaccination or challenge, and to also see if NAbs levels were associated with the liver fluke burden in cattle after experimental parasite infection.

## 2. Materials and Methods

### 2.1. Experimental Animals, Vaccination and Sampling

Three separate vaccine trials were analysed here (studies A, B, C) and the details of each trial are shown in Supplementary Table S1. Serum samples from the entirety of study A was used, which were taken from Angus and Angus Hereford crosses all at 6 months of age after random allocation into groups with similar body weight ranges. Prior to commencement, all animals were confirmed negative for *F. hepatica* infections by faecal egg counts (FEC), the liver fluke coproantigen ELISA (BioX Diagnostics, Rochefort, Belgium) and serological ELISA (BioX Diagnostics) following the manufacturer’s instructions and as described [34,35]. Throughout all studies, animals had ad libitum access to pasture and were retained on a single paddock that did not contain waterbodies for the intermediate snail host to propagate.

Initially, a vaccinated group and corresponding control group from study A (*n* = 6 per group) were assessed for their natural antibody levels and their specific humoral responses. These animals were subcutaneously injected on either side of the lateral aspect of the neck on day 0 and day 28, with a total of 1 mL of vaccine. The vaccinated group was co-administered with 200 µg of native *F. hepatica* GST and 200 µg of a tegument antigen solubilised in PBS (137 mM NaCl, 2.7 mM KCl, 10 mM Na_2_HPO_4_, 1.8 mM KH_2_PO_4_, pH 7.4) and mixed 1:1 with Freund’s complete adjuvant (FCA) (Sigma-Aldrich, St. Louis, MO, USA) on day 0 and Freund’s incomplete adjuvant (FIA) (Sigma-Aldrich) on day 28. The control group was vaccinated with PBS solution in FCA then FIA on days 0 and 28, respectively. Animals were then challenged 6 weeks after the first vaccination (day 42) with 350 viable metacercariae (Oberon strain, Invetus Pty Ltd., Armidale, Australia) and necropsied 12 weeks post-infection on day 125 when liver pathology was scored, and fluke burdens and efficacy were determined as described [34,36]. Serum samples were taken and assessed from day 0 (naïve pre-vaccination), day 42 (pre-challenge and post-vaccination), day 84 (6 weeks post-challenge) and day 125 (12 weeks post-challenge).

Secondly, to increase the statistical power, a larger dataset of experimentally infected control group animals (*n* = 19) from different vaccination trials (studies A, B and C) were collated and analysed for their humoral responses and fluke burdens. Animals experimentally infected had serum samples assessed from the day of challenge (naïve) and 6 weeks after challenge (infected). All experimentally infected animals were sacrificed about 12 weeks after challenge for fluke burden determination. It should be noted that the experimentally infected animals are combined control groups from different studies A, B and C that received varying metacercarial loads (depicted in Supplementary Table S1). This is analogous to groups of animals with varying levels of natural infection, where there is no fixed metacercarial intake numbers and thus infection levels would vary to an unknown degree amongst animals.

Invetus Pty Ltd. processed the bloods and completed the FECs and necropsies as described [34,35]. Animal welfare and permits for these studies are shown in Supplementary Table S1.

### 2.2. Acquisition of Parasite Whole-Worm Extract and Purification of Parasitic Glutathione S-Transferase

Adult liver flukes were obtained from naturally infected bovine livers at a local Victorian abattoir. Flukes were washed at room temperature in PBS three times and stored at −80 °C until processed for either whole-worm extract (WWE) or native GST isolation. WWE was prepared as described [37]. Briefly, flukes were snap frozen in liquid nitrogen and homogenized by mortar and pestle, and subsequently washed in RIPA buffer (20 mM Tris-HCl [pH 8], 150 mM NaCl, 0.5 mM ethylenediaminetetraacetic acid (EDTA), 1% (*v*/*v*) Nonidet P-40, 0.1% (*w*/*v*) sodium deoxycholate, 0.05% (*w*/*v*) sodium dodecyl sulfate (SDS), 1% (*v*/*v*) Triton X-100). The WWE extract was then clarified by centrifugation at 100,000× *g* for 1 h and the supernatant was collected, filtered through a 0.22 µm filter and passively dialysed (3.5 kDa MWCO) into RIPA dialysis buffer (RIPA buffer without SDS and with 0.05% (*v*/*v*) Nonidet P-40 and 0.01% (*w*/*v*) sodium deoxycholate). Native GST was purified via glutathione (GSH) affinity resin (GE Healthcare, Chicago, IL, USA) following the manufacturer’s instruction with adaptations from [38]. Briefly, flukes were homogenized within wash buffer (10 mM EDTA, 2 mM PMSF, 0.15 M NaCl, 50 mM Tris-HCl [pH 8], 0.5% Triton X-100, pH 7.3) and the homogenate clarified by centrifugation at 12,000× *g* for 30 min. The subsequent lysate was incubated overnight at 4 °C with 1 mL of pre-equilibrated GSH resin with head-over-tail mixing. Lysate slurry was then decanted into a column with the flow through rate under gravity pressure and the resin was then washed with 10 column volumes of wash buffer and eluted with 8 mL of elution buffer (50 mM Tris-HCl [pH 8], containing 10 mM GSH). Purified GST was then buffer exchanged into PBS using Amicon^®^ Ultra-15 3K filter units (Millipore, Burlington, MA, USA). Protein was quantified using the Pierce^TM^ BCA (bicinchoninic acid) Protein Assay Kit (Thermo Fisher Scientific, Scoresby, Australia) with BSA as the reference.

### 2.3. Indirect ELISA Analysis of Natural Antibodies and Specific Antibody Responses

The reactivity of IgG and IgM to exogenous antigen KLH (Sigma-Aldrich) was optimised using a pool (*n* = 12, Study A) of sera collected from parasite-naïve cattle prior to infection or vaccination. A flat bottom 96-well medium-binding ELISA plate (Greiner Bio-one, Kremsmünster, Austria) was coated with KLH (1 µg/mL) in carbonate bicarbonate buffer (28.6 mM Na_2_CO_3_, 71.5 mM NaHCO_3_, pH 9.6) overnight at 4 °C. Each of the following incubations was completed at 37 °C and preceded with 5 washes of PBS-T (PBS, 0.05% (*v*/*v*) Tween 20). All wells were blocked for 2 h with 200 µL of 2.5% (*w*/*v*) fish gelatine from cold water fish (Sigma-Aldrich) dissolved in PBS-T. To determine the optimal serum dilution for NAb assessment, plates were incubated for 1 h with duplicate aliquots of 100 µL of the pooled serum in two-fold serial dilutions, from 1:40 to 1:2560, diluted in blocking buffer. Plates were subsequently incubated for 1 h with secondary antibodies; either sheep HRP anti-bovine IgM (Bio-Rad, Hercules, CA, USA) or rabbit HRP anti-bovine IgG (Bio-Rad), both diluted 1:5000 in blocking buffer. TMB substrate (1-Step™ Turbo TMB-ELISA, Thermo Fisher Scientific) was then added (100 µL/well) and incubated in the dark for 10 min followed by the addition of 100 µL/well of 1 M HCl. Absorbance was measured at 450 nm on the xMark^TM^ Microplate Spectrophotometer (Bio-Rad).

Natural antibody responses for individual sera were then analysed under the same conditions with the optimal serum dilution of 1:200, determined from the dilution gaining an optical density (OD) 50% of that of the maximum OD observed. SpAb responses were simultaneously analysed in the same assay, with the equivalent incubations and dilutions to the NAb assay. The vaccinated and control group in study A was tested for SpAb responses towards GST on plates coated with 5 µg/mL of native GST, and the experimentally infected animals in studies A, B and C were tested for their response to WWE on plates coated with 5 µg/mL of WWE. All samples were assessed in duplicate on the same plate and each assay was repeated, resulting in four measurements for each sample. Negative controls were set up in duplicate, where blocking buffer replaced antigen (serum background) and where blocking buffer replaced serum (secondary background reactivity). All assays have a cut-off value, calculated from the mean OD plus 3 standard deviations from the mean OD of blank wells (blocking buffer in replace of antigen, sera and secondary). The pooled naïve sample used for assay optimisation was replicated in duplicate on each plate towards each antigen. Results are displayed as the mean (*n* = 4) index of the OD, which is calculated as the fraction of the adjusted sample OD divided by the adjusted OD of the pooled naïve sample towards the same antigen or extract, where the adjusted OD is the OD of the sample after subtracting the mean OD of the serum background (no antigen).

### 2.4. Statistical Analysis

Normality of the unavoidably small sample size was determined through histograms and Q-Q plots (Origin 2019 64 Bit). Two datasets within the experimentally infected animals had outliers removed (after further analysis through box plots) so normality could be assumed. Differences in vaccine efficacies based on fluke burdens and fluke wet weights were determined using one-way ANOVA as were differences in antibody levels since all were considered parametric. Linear regression analysis was determined using Pearson’s correlation, with results considered significant with a *p*-value < 0.05 and trends noted with a *p* < 0.15.

## 3. Results

### 3.1. Liver Fluke Burdens and Fluke Wet Weights in Control and Vaccinated Cattle

**Figure 1:**
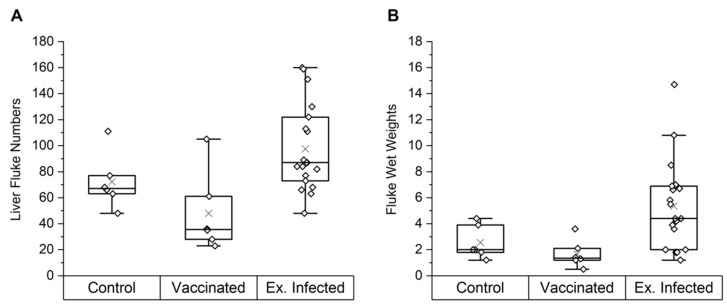
Final fluke parameters for the control and vaccinated groups in study A and experimentally infected cows in studies A, B and C. Liver fluke numbers (**A**) and fluke wet weights (**B**) are shown. Results are displayed as quartile box plots with a median line and a mean cross (×). Animals in the vaccinated group received two co-administered doses of native *F. hepatica* GST and a tegumental antigen in Freund’s complete adjuvant (FCA) then Freund’s incomplete adjuvant (FIA) 4 weeks apart. Control animals were vaccinated with saline in FCA then FIA. Both the vaccinated and control group were then challenged with liver fluke metacercariae 6 weeks after the first vaccination and sacrificed 12 weeks after challenge. No significant difference in mean fluke numbers was noted between the control and vaccinated group (*p* = 0.14). Experimentally infected animals were all sacrificed 12 weeks post-challenge with liver fluke metacercariae loads according to Supplementary Table S1. Abbreviations: Ex. Infected, experimentally infected animals.

To investigate if NAbs have an effect on parasite infections in cattle, the fluke burdens were determined from a group of cattle vaccinated with a combination of native *F. hepatica* GST and a tegumental antigen (*n* = 6), its control group (*n* = 6) (study A), and 2 other groups of experimentally infected control animals (study B: *n* = 6, and study C: *n* = 7), resulting in a total of 19 experimentally infected cattle. Figure 1 displays the final liver fluke numbers and total wet weights of the control, vaccinated and experimentally infected animals in studies A, B and C, with individual parameters also reported (Supplementary Table S2). In study A, the vaccinated group showed a non-significant 33% vaccine efficacy (i.e., reduction in fluke burden) compared to its control group. However, the means of the control (72 ± 21) and the vaccinated (48 ± 31) animals were not significantly different (*p* = 0.14, one-way ANOVA). Within the same trial, the tegumental antigen was administered alone in a separate group and showed no protective efficacy (reported in [36]); for this reason, GST is the antigen of focus in this study.

Four of the six vaccinated animals showed lower fluke burdens than the lowest burden within the control group; burdens of 23, 28, 35 and 36 compared to 48, respectively. A vaccinated animal (V1) appeared to be a vaccine non-responder, containing a high number (105) of flukes, contributing to the large range (23–105) of fluke burdens within the vaccinated group. Across all 3 studies, the 19 experimentally infected control animals had a large range of burdens (48–160) and a mean ± SD of 98 ± 33.

### 3.2. Optimisation of NAb Assays

Pooled parasite-naïve sera (*n* = 12, study A) were selected for the development of the NAb assay. These pooled sera had higher reactivity for both IgG and IgM towards KLH compared to the no antigen control (Supplementary Figure S1). The optimal serum dilution to identify NAb reactivity to KLH was determined from the serum log2 value at approximately 50% of the maximum OD (OD50). A 1:200 serum dilution factor (or 7.8 log2 value) was chosen as the optimal serum dilution for both the IgG and IgM assay, as it was the middle value between the OD50 for IgG and IgM. This dilution was subsequently used to identify the SpAb response towards parasite GST in the vaccinated and control groups, and the SpAb response towards WWE in the experimentally infected animals.

### 3.3. Fluctuations in Mean NAb and SpAb Levels throughout the Vaccination Trial

**Figure 2:**
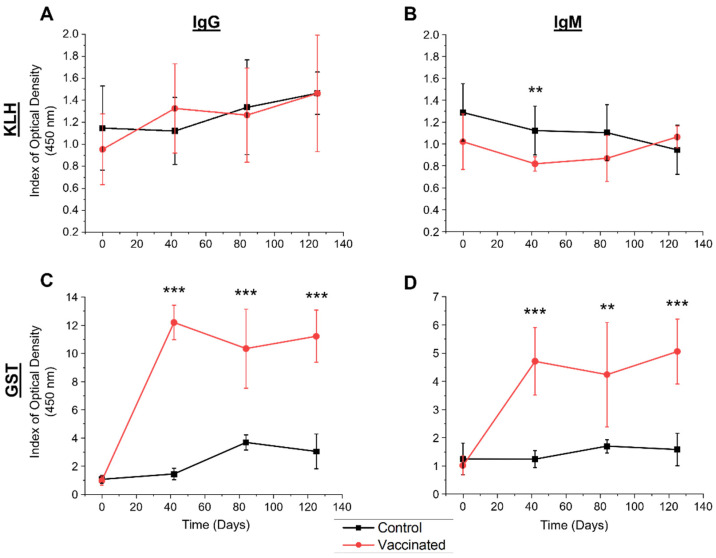
Humoral responses in study A to KLH and GST in the vaccinated and control groups as determined by ELISA. Bovine serum samples were analysed from days 0 (naïve, pre-vaccination), 42 (pre-challenge, post-vaccination), 84 (6 weeks post-challenge) and 125 (12 weeks post-challenge) for the control (black squares) and vaccinated (red circles) groups. Displayed are the indexes of the optical density for IgG (**A**,**C**) and IgM (**B**,**D**) for the natural antibody response towards KLH (**A**,**B**) and specific antibody response towards native *F. hepatica* GST (**C**,**D**), assessed four independent times. Results are expressed as the mean index of optical density (450 nm) for the group (*n* = 6) +/− the standard deviation (error bars) of the mean indexes. The vaccination group received native GST and a tegumental antigen at day 0 and 28 in Freund’s complete adjuvant (FCA) then Freund’s incomplete adjuvant (FIA), and the control group received saline injections in FCA/FIA. Both groups were then challenged on day 42 with liver fluke metacercariae and sacrificed 12 weeks after challenge. Any significant differences in means are noted (one-way ANOVA, ** = *p* < 0.001, *** = *p* < 0.001). Abbreviation: GST, native glutathione *S*-transferase.

The indirect ELISA detected bovine serum IgG and IgM binding indexes over the course of trial A (days 0, 42, 84, and 125) from animals vaccinated with native GST (plus a tegumental antigen) in FCA/FIA and from the control group vaccinated with PBS in FCA/FIA. The mean SpAb responses towards native GST and the NAb binding towards KLH are shown in Figure 2. A significant difference of mean indexes was observed between the groups for the IgM NAb response at day 42 following vaccination (Figure 2B), with a tight index range within the vaccinated group of 0.59 and a lower mean response compared to the control group. No significant difference was observed for the IgG NAb response between the control and vaccinated groups. The mean index for specific anti-GST responses within the vaccination group was significantly higher after vaccination (day 42) and throughout the trial (day 84 and day 125) compared to the control group for IgG (Figure 2C) and IgM (Figure 2D). 

**Figure 3:**
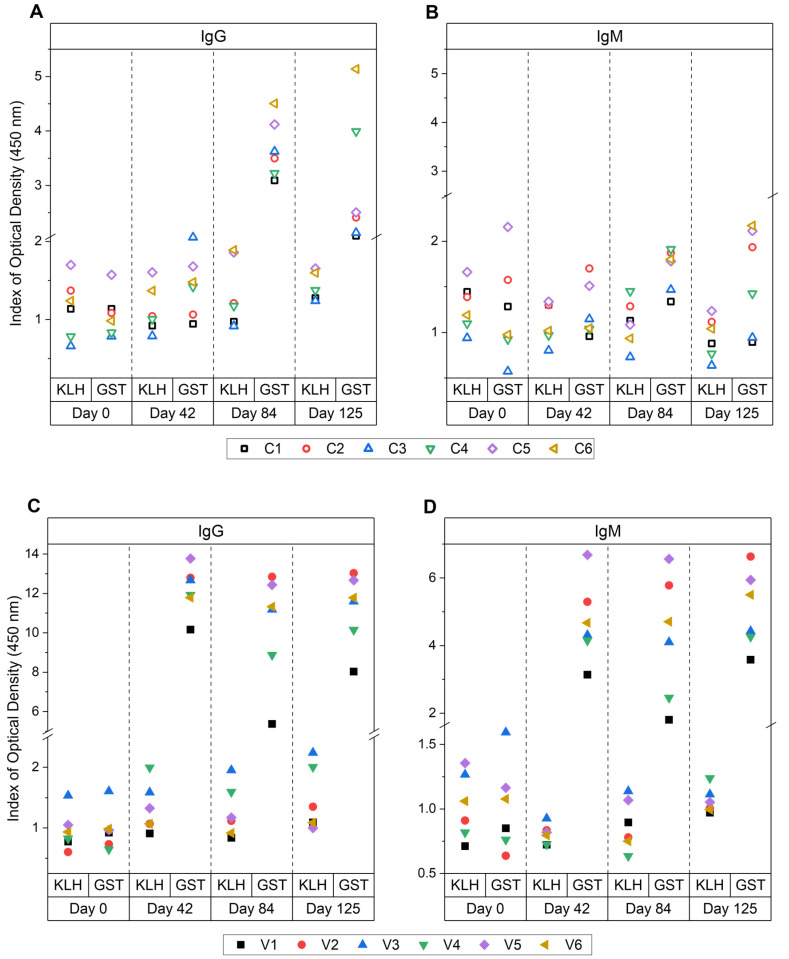
Individual antibody responses for animals within the control and vaccinated group in study A. ELISA responses to antigens KLH and native glutathione *S*-transferase (GST) from bovine serum samples from day 0 (naïve, pre-vaccination), day 42 (pre-challenge, post-vaccination), day 84 (6 weeks post-challenge) and day 125 (12 weeks post-challenge). Control group IgG (**A**), control group IgM (**B**), vaccinated group IgG (**C**) and vaccinated group IgM (**D**) responses are shown. Results are reported as the mean index (*n* = 4) of optical density (450 nm) for the individual cows. The vaccination group received native GST and a tegumental antigen at day 0 and 28 in Freund’s complete adjuvant (FCA) then Freund’s incomplete adjuvant (FIA), and the control group received saline injections in FCA/FIA. Both groups were then challenged on day 42 with liver fluke metacercariae and sacrificed 12 weeks after challenge. All cut-off values fell below 0.07 for all assays.

In Figure 3, the mean reactive SpAb and NAb indexes are shown for the individual animals in the control and vaccinated groups against each antibody isotype throughout the vaccination trial. The SpAb production towards GST was established after vaccination and sustained throughout the trial for IgG (Figure 3C) and IgM (Figure 3D). There was variation in the level of SpAb responses amongst the vaccinated cattle. Animal V1 showed relatively lower SpAb IgG and IgM levels compared to animals V2 and V5, which were the highest responding animals for specific IgG and IgM post-vaccination at day 42 and post-challenge days 84 and 125. A notable observation of SpAb production in the control group was in animal C6 which had the highest IgG production after challenge at days 84 and 125 (Figure 3A).

**Figure 4:**
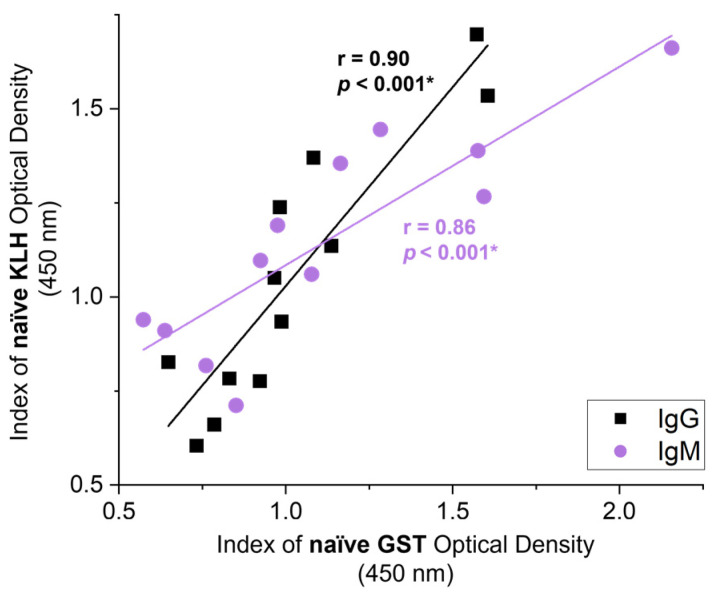
Correlations between the naïve antibody responses for KLH and native GST. Summated naïve serum samples from the control (*n* = 6) and vaccination (*n* = 6) groups in study A collected on day 0 before vaccination or challenge and analysed to assess NAb responses to KLH and native glutathione *S*-transferase (GST). Correlation coefficients of the mean index (*n* = 4) of optical density (450 nm) for IgG NAb (black) and IgM NAb (purple) responses towards KLH and native GST are shown. Pearson’s correlation coefficient (r) and the corresponding significance value are noted on the graph next to the regression line. Significant values (*p* < 0.05) are bolded and noted with an asterisk (*).

We assessed correlations between the KLH-binding NAbs with NAbs binding to parasite GST prior to exposure to either antigen, to assess if NAbs have the potential to cross-react and bind to liver fluke proteins. Summating both groups’ responses to KLH and GST (Figure 4) at day 0 (i.e., the 12 naïve serum samples before vaccination in study A) showed significant correlations between the two responses for both IgG (r = 0.90, *p* < 0.001) and IgM (r = 0.86, *p* < 0.001). This suggests that NAbs are capable of binding to parasite GST. 

### 3.4. Relationships between Naïve NAb and Vaccine-Induced SpAb Production

**Figure 5:**
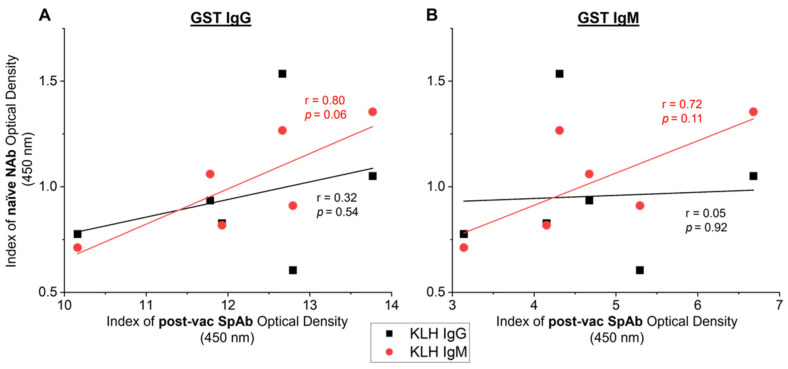
Relationship between specific antibodies (SpAbs) induced post-vaccination and the naïve natural antibodies (NAbs) in cows from study A (*n* = 6). SpAb response towards native glutathione *S*-transferase (GST) IgG (**A**) and GST IgM (**B**) post-vaccination (post-vac) (day 42) were correlated to the naïve pre-vaccination (day 0) NAb response to IgG KLH (black squares) and IgM KLH (red circles). Linear regression lines are displayed for each SpAb and NAb relationship. Pearson’s correlation coefficients (r) and the corresponding significance value are noted on the graph next to the regression line. Results are reported as the mean index (*n* = 4) of optical density (450 nm).

Pearson’s correlations were used to assess the association between naïve NAb responses and SpAb production after vaccination with native GST. The vaccinated animal with the highest (V5) and the lowest (V1) day 0 naïve KLH-reactive IgM NAb responses subsequently showed the highest (V5) and lowest (V1) IgG and IgM SpAb levels to GST after native GST vaccination at day 42, as depicted in Figure 3C,D. Nevertheless, there was no significant correlations between the naïve day 0 anti-KLH IgM NAbs or anti-KLH IgG responses and the vaccine-induced anti-GST IgG or IgM SpAbs from day 42 (Figure 5). However, the naïve IgM NAb levels may reflect the subsequent SpAb production post-vaccination, as there is a strong trend in the associations between KLH-binding IgM NAbs to both anti-GST SpAb IgG (Figure 5A: r = 0.80, *p* = 0.06) and IgM (Figure 5B: r = 0.72, *p* = 0.11). The lack of significance is potentially a by-product of the limited sample size. 

### 3.5. Correlations between NAb and SpAb Levels to Final Liver Fluke Burdens and Wet Weights

No significant correlations were seen in the control or vaccinated groups between either NAb or SpAb production and the final liver fluke burdens (Table 1). Conversely, significance was noted in the vaccinated group between fluke weights and the anti-GST SpAb responses post-vaccination and onwards for IgG (Day 42: r = −0.83, *p* = 0.04, Day 84: r = −0.82, *p* = 0.04, Day 125: r = −0.81, *p* = 0.04) and at day 42 for IgM (r = −0.85, *p* = 0.03). Furthermore, following vaccination the SpAb responses showed strong negative trends to the final fluke burdens, notably following vaccination and on the day of infection for both IgG (r = −0.77, *p* = 0.07) and IgM (r = −0.75, *p* = 0.08) with trends not observed at the same time points in the control group, indicating a potential link between vaccine-induced anti-GST antibodies and final fluke levels. No notable trends were observed for NAb levels and liver fluke burdens or wet weights in the vaccinated group. However, the control group did show a negative trend between final fluke burdens and the anti-KLH IgM responses post-challenge (Day 84: r = −0.76, *p* = 0.08), with the group showing no trends to fluke weights.

### 3.6. NAb and SpAb Levels to Fluke WWE before and after Experimental Infection

**Figure 6:**
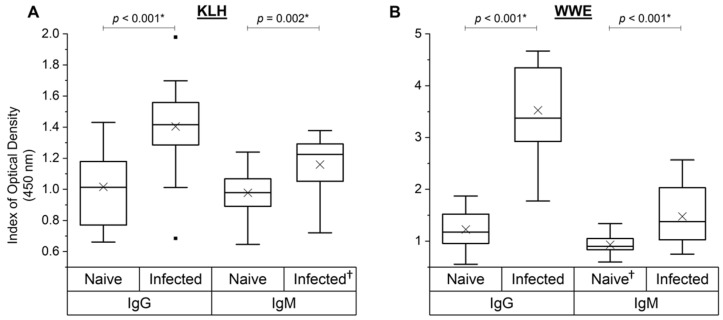
Analysis of natural and specific antibody levels for experimentally infected animals from cows in studies A, B and C (*n* = 19). KLH-binding IgG and IgM (**A**) and liver fluke whole-worm extract (WWE)-binding IgG and IgM (**B**) on the day of experimental liver fluke infection (naïve) and 6 weeks post-infection (infected) were assessed. Data are presented as the mean index (*n* = 4) of optical density (450 nm) in quartile box plots with a median line and a mean cross (×) for the experimentally infected animals. Any significant differences (*p* < 0.05) in means are noted with an asterisk (*) in the graph (one-way ANOVA). ^†^: datasets that had an outlier removed (*n* = 18).

Sera from the 19 control animals from studies A, B and C that were experimentally infected with liver fluke metacercariae were analysed at the day of challenge (naïve) and 6 weeks after challenge (infected) for their NAb responses towards KLH and SpAb response to liver fluke WWE. Parasite exposure did influence the overall NAb and SpAb anti-WWE levels (Figure 6). Unsurprisingly, there was a strong increase in SpAb production to WWE after parasite challenge for both IgG (*p* < 0.001) and IgM (*p* < 0.001) (Figure 6B). Interestingly, NAb binding to KLH also increased after infection for both IgG (*p* < 0.001) and IgM (*p* = 0.002) (Figure 6A). There was no direct association between the KLH-binding NAb and NAbs to WWE at the naïve time point (IgG: r = 0.20, *p* = 0.42; IgM: r = 0.26, *p* = 0.29; data not depicted in a figure).

### 3.7. Correlations between Naïve NAbs and Specific WWE Production Post-Infection

**Figure 7:**
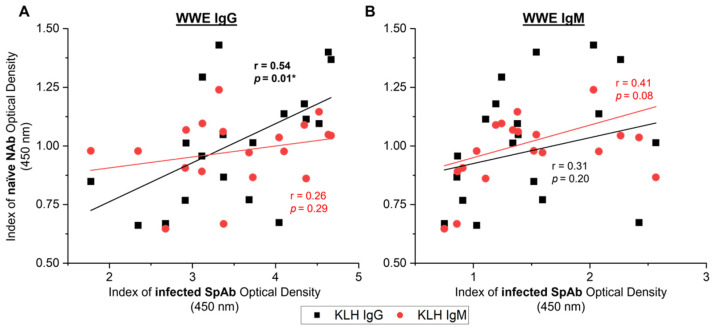
Relationship between naïve natural antibodies (NAbs) from the day of challenge (naïve) and liver fluke specific antibodies (SpAbs) to whole-worm extract (WWE) 6 weeks post-infection (infected) in the experimentally infected cows from studies A, B and C (*n* = 19). SpAb response for WWE IgG (**A**) and WWE IgM (**B**) 6 weeks after infection were correlated to the naïve NAb response to IgG KLH (black squares) and IgM KLH (red circles). Linear regression lines are displayed for each SpAb and NAb relationship. Pearson’s correlation coefficients (r) and the corresponding significance value are noted on the graph next to the regression line. Results are reported as the mean index (*n* = 4) of optical density (450 nm). Significant values (*p* < 0.05) are bolded and noted with an asterisk (*).

To investigate if underlying NAb reactivity can predict the SpAbs produced to whole-parasite-protein extract after parasite challenge, the naïve KLH-binding levels in all 19 animals on the day of challenge were correlated to WWE reactive antibodies 6 weeks post-infection (Figure 7). The naïve IgG KLH-binding NAb reactivity showed a significant positive correlation to the production of anti-WWE IgG SpAbs after infection (Figure 7A: r = 0.54, *p* = 0.01). However, this was not observed with naïve IgM KLH-binding NAb and anti-WWE IgG post-infection (Figure 7A). A potential weak–moderate trend was seen between specific WWE IgM production and anti-KLH IgM (Figure 7B: r = 0.41, *p* = 0.08), but not for anti-KLH IgG.

**Figure 8:**
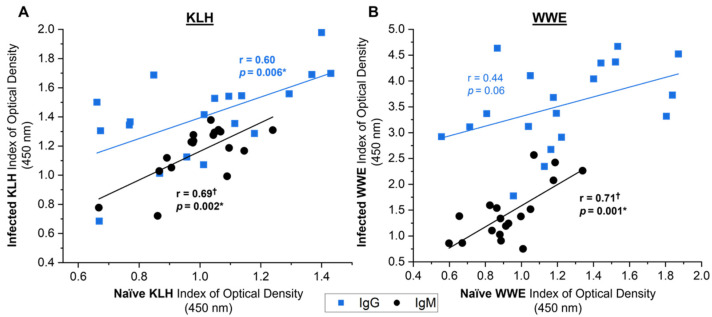
Relationship between antibody responses from the day of infection (naïve) and 6 weeks post-infection (infected) towards the same antigen (KLH) or extract (WWE) in the experimentally infected cows from studies A, B and C (*n* = 19). Antibody responses to KLH (**A**) and whole-worm extract (WWE) (**B**) for IgG (blue squares) and IgM (black circles) are shown. Linear regression lines display the antibody response relationship for KLH or WWE between the naïve and infected time points. Pearson’s correlation coefficients (r) and the corresponding significance value are noted on the graph next to the regression line. Results are reported as the mean index (*n* = 4) of optical density (450 nm). Significant values (*p* < 0.05) are bolded and noted with an asterisk (*). ^†^: datasets that had an outlier removed (*n* = 18).

In contrast, multiple significant associations were observed between the naïve NAb responses towards the subsequent responses of the same isotype and antigen (KLH) or extract (WWE) 6 weeks post-infection in the experimentally infected animals (Figure 8). The naïve KLH-binding NAb response showed moderate-strong correlations to the subsequent KLH-binding NAb following infection (Figure 8A IgG: r = 0.60, *p* = 0.006; IgM: r = 0.69, *p* = 0.002). Notably, the naïve NAbs reactive to WWE had a strong significant associations to the anti-WWE SpAb response 6 weeks post-infection for the IgM isotype (Figure 8B: r = 0.71, *p* = 0.001) and only a trend was observed for IgG (Figure 8B: r = 0.44, *p* = 0.06). Overall, this suggests that the subset of naïve NAbs that bind to WWE prior to liver fluke exposure could be associated with the anti-WWE SpAb production 6 weeks post-infection, making the naïve WWE NAb response a potential indicator of specific anti-WWE production.

### 3.8. Antibody Correlations to Final Liver Fluke Burdens and Wet Weights after Experimental Infection

The KLH-binding IgG and IgM NAb levels from the day of infection (naïve) or 6 weeks post-infection (infected) did not show significant associations to fluke burdens or wet weights in the experimentally infected cows from study A, B and C (Table 2). However, one notable positive trend was observed for anti-KLH IgM post-infection and fluke burden (r = 0.43, *p* = 0.07). Interestingly, the naïve WWE-reactive IgM levels did show a significant moderate positive relationship (r = 0.56, *p* = 0.02) to the cows subsequent final liver fluke burdens. No links were observed between any SpAb response and fluke wet weights, or the specific WWE IgG responses and fluke burdens in the experimentally infected animals.

## 4. Discussion

Despite numerous vaccine trials testing a range of different antigens, there is no current consistently effective vaccine against the liver fluke *F. hepatica* [12,15,39]. In recent years, NAbs have been shown to play pivotal roles in the first line of defence against pathogens. However, the effect of NAbs on the production of specific antibodies due to vaccination is poorly understood and unknown during liver fluke infections in cattle. Here, we present the potential roles of NAbs by determining possible correlations between NAbs and the production of SpAbs and helminth parasite burdens after experimental infection and targeted vaccination.

Selective breeding against non-vaccine-responding animals, for high specific antibody production, by analysing their NAb responses prior to vaccination could potentially result in a consistent reduction in fluke burdens and diminish infection severity in a herd of animals. Bovine NAbs are heritable and it is therefore feasible to undergo selective breeding programs for high NAb levels [40]. NAbs are under genetic control as they are germline encoded, and bovine natural IgG and IgM binding KLH antibodies from both plasma and milk have been shown to be heritable and could be exploited as a biomarker in breeding programs [27,30,41,42,43,44]. A genome-wide association study of 925 Holstein cows concluded that plasma KLH-binding IgM NAbs is moderately heritable (0.31) and generally has a higher heritability than IgG NAbs (0.27) [41], with comparable values noted in other bovine studies [27,30,42,43]. There are no studies evaluating ruminant NAbs in breeding programs; however, chickens can be divergently selected for high NAb levels to improve disease resilience [24,26,45]. After 4 generations, chickens selected for high KLH-binding NAbs at 16 weeks of age had a greater survival rate of 91% against experimental avian pathogenic *E. coli* infections, compared to 76% survival in the low-NAb line [24]. In cattle, the incidence rates of displaying clinical mastitis is reflected in their NAb levels, as Canadian Holstein cows with increased KLH-binding IgM NAbs had a decreased risk of clinical mastitis [27], but this phenomenon is yet to be exploited and evaluated in a selective breeding program. Lowering parasitic infection severity in livestock through selective breeding of antibody levels has been shown in *Teladorsagia circumcincta* infections in sheep. Selecting rams for higher plasma IgA levels over 10 generations reduced worm growth and worm fecundity at a quicker rate compared to selection based on FEC; FEC dropped by 85% using IgA as a selective marker, compared to the drop of 50% for selection using FEC after 7 generations [46]. This effect results from the stronger heritability of IgA over FEC and the direct affects that IgA has upon worm growth [46]. While NAbs have an unknown effect on helminth parasites, using bovine NAbs as a breeding biomarker is feasible and attractive.

The present study shows evidence that bovine NAb levels can mirror the production of SpAbs after challenge and potentially after vaccination. Increased initial levels of underlying KLH-reactive IgM indicated a greater humoral response towards the GST antigen, with trends to higher production of anti-GST IgG and IgM. Furthermore, this study identified a moderate to strong significant positive association between naïve IgG KLH-binding NAbs and the production of specific IgG toward WWE 6 weeks after infection, indicating that NAb levels can allude to the subsequent production of parasite specific IgG produced after infection. The literature depicts different relationship types between NAbs and SpAbs that appear to vary with the antigens generating the SpAb response and the antigen used to define the NAb levels. After two generations of high- and low-divergent selection of KLH-binding NAbs, chickens were immunised with different immunogens known to stimulate a Th1 (avian tuberculin purified protein derived of *Mycobacterium avium*; PPD) and a Th2 (human serum albumin; HuSA) response [26]. Interestingly, the dynamics between NAbs and SpAbs were vaccine-antigen dependent, with indications of a positive NAb relationship between the Th2 driving immunised antigen and no difference in SpAb levels in high and low lines when vaccinated with the Th1 antigen [26]. Therefore, the authors postulated that chickens with higher NAb levels are more readily influenced by Th2-stimulating antigens as the humoral response is evoked [26]. Here, cattle were vaccinated with native GST in the Freund’s adjuvant system, a known strong Th1 stimulating adjuvant [47], and, in contrast to the aforementioned chicken study, showed a positive trends between IgM NAbs and specific anti-GST IgG and IgM within the Th1 adjuvant generated immune environment. After liver fluke challenge, which is known to modulate the bovine immune system towards a Th2 immune response generated by the parasite [48], there was a significant positive relationship between IgG NAbs and IgG SpAbs towards whole-parasite extract. This suggests that that bovine NAbs have the potential to positively reflect SpAb production under either a humoral or cell mediated immune bias. Further investigation is warranted in a larger sample size to continue exploring the influence of bovine NAbs and vaccine-induced SpAb production.

Previous investigations into livestock NAb and SpAb production did not reveal significant correlations. Using 451 Canadian Holstein cows vaccinated with egg white lysozyme (to induce a humoral response and not to create protection against disease), cows did not show genetic SpAb correlations to KLH-binding NAbs. Furthermore, goats vaccinated with the commercial Bravoxin^®^ 10 (MSD Animal Health, Kenilworth, NJ, USA) vaccine containing inactivated clostridial antigens and the Th2 stimulating adjuvant alum [49] did not show any correlations between NAbs binding 2,4,6-trinitrophenol (TNP) and SpAb production [50]. TNP was used to define NAb levels in that study as it showed the highest NAb reactivity in a pre-screen of various exogenous antigens. However, it would have been interesting to simultaneously determine if SpAb correlations existed between the most frequently used antigen, KLH. Negative associations between NAbs and SpAbs have been observed in tortoises after immunisation with ovalbumin (OVA), potentially from NAbs displaying cross-reactivity to OVA and hiding epitopes [51]. The present study showed differing results, as KLH-binding NAbs were potentially cross-reactive with GST when naïve sera were assayed against both antigens, suggesting a potential for epitope hiding (i.e., neutralisation of epitopes), but a subsequent positive trend to GST was still observed after vaccination for IgM NAb and anti-GST IgG and IgM production. It is possible that varied effects of NAb cross-reactivity is based on the host species, as reptiles rely more heavily on NAbs to clear pathogens as they have a slower and less potent SpAb production compared to mammals [51]. Ultimately, it is likely that the relationship between NAb and SpAb responses is multifactorial and dependent on; the antigen used for NAb definition, the immunised antigen, and host species.

While assessing KLH antibody binding is important because of its heritability and for consistency across studies, NAb distinction using a conserved molecule in the target pathogen could be invaluable. KLH-binding NAbs were used in the present study; however other conserved pathogen recognition molecular patterns (PAMP) or danger associated molecular patterns (DAMPs) can be used to define NAbs. Previous studies have shown that individual cattle show differences in both plasma and milk samples in their NAb reactivity to other PAMPs; lipopolysaccharide (LPS), lipoteichoic acid (LTA) and peptidoglycan (PGN), suggesting a diverse NAb population within animals [29]. However, when grouped in high and low KLH-binding NAb pools, cattle do retain high and low reactivity towards the above PAMP molecules [28]. Nevertheless, when different KLH-reactive pools were assessed for reactivity towards *E. coli* and *Salmonella* typhimurium, there was no difference observed in divergent NAb pools for IgG towards *E. coli* but there was for *S.* typhimurium [28]. It was suggested that the difference in binding could be based on the differences in the bacterium’s expression of LPS; with *E. coli* and *S.* typhimurium expressing rough and smooth LPS, respectively [28]. Ultimately these results suggest that the complex NAb repertoire may not be entirely defined by assessing the response towards a single exogenous antigen (such as KLH) and that antigen selection is key in defining the NAb population that interacts with the pathogen of interest. Therefore, identification of the NAb subset within the heterogeneous NAb population that interacts with liver fluke proteins could take place using a conserved molecule or molecules within the fluke’s WWE.

Despite prior evidence that NAbs can subdue virus and bacterial infections and participate in ADCC in other systems [17,19,52], this study showed that NAb levels are elevated after infection and higher NAb IgM levels are linked to increased liver fluke burdens in experimentally infected cattle. While the mechanisms of in vivo killing of flukes are unknown, in vitro studies show that fluke death can be mediated by ADCC [9,10,53], however the specific antigen and antibody isotypes involved in fluke ADCC are still uncertain. NAbs can participate in ADCC attack in vitro via interactions with natural killer cells, as shown by incubation of human natural killer cells with IgG NAbs specific to galactose-alpha-1,3-galactose and the subsequent lysis of porcine target cells [52]. Therefore, NAbs have the capability of initiating the ADCC mechanism that can potentially damage flukes and are secreted by B1 cells located in the peritoneal cavity, where the NEJs reside while they traverse to the liver [21,54]. In this study, the experimentally infected animals from studies A, B and C were naïve to infection at the initial sampling point, implying that the reactivity seen at the naïve time point towards WWE is likely due to NAb reactivity. However, the interesting significant positive relationship between bovine IgM binding WWE prior to infection and the final fluke burdens suggests that natural IgM antibodies may not hinder fluke survival within the host.

It is possible that juvenile liver flukes could utilise the surface binding of NAbs within the peritoneal cavity to protect the tegument from the binding of other antibody isotypes that trigger ADCC attack. The tegument interface between the parasite and host is a highly glycosylated layer containing glycostructures [55,56,57]. Accordingly, with the known NAb polyreactivity towards conserved structures, it could be postulated that IgM NAbs can potentially bind to the fluke’s glycosylated tegument, protecting them from the onslaught of the host immune system. IgM has been observed to attach to the NEJs surface through immunohistochemical staining of 4-week-old juveniles in ovine hepatic parenchyma and it is proposed that this binding can block eosinophil attachment to the fluke’s surface and limit degranulation as eosinophils lack the Fcµ receptor that promotes IgM function [58]. Eosinophils have a known function in ADCC, and it has been shown that eosinophils are beneficial to the rat host during *F. hepatica* infection, as they limit the production of Th2 cytokine IL-10 [59]. While the role of eosinophils in fluke infections is incomplete, blocking effective docking of eosinophils to antibodies bound to the fluke surface could reduce parasite killing, which is potentially evidenced here by the positive NAb IgM association with fluke burdens. Further investigation is required in other liver fluke-susceptible species and a larger sample size to determine if host NAbs play a role in liver fluke immune evasion and if NAbs can potentially be used as a breeding selection marker while vaccine development is underway.

## 5. Conclusions

Natural antibodies are an innate immune response capable of protecting production animals against pathogens and can be a marker for an animals’ health status or disease resilience. The role of NAbs during parasitic helminth infections has not been previously investigated. The protective immune mechanism against *Fasciola* spp. is not fully understood and currently tested vaccine molecules have not stimulated a sufficient and reproducible protective response. Herein, we investigated if bovine NAb levels can reflect the subsequent vaccine-induced specific antibody responses towards glutathione *S*-transferase following vaccination and if NAbs link to final fluke numbers after experimental infection. It was found that NAbs reacting to the exogenous KLH antigen only showed trends to and was not significantly correlated to vaccine-induced SpAb production and that NAbs had no significant links to liver fluke burdens following vaccination. However, the vaccinated group, while having a limited sample size, showed an interesting positive trend between underlying IgM NAbs and vaccine-induced anti-GST IgG, which is worth further exploration in a larger dataset. Cows that were experimentally infected with liver fluke showed a significant positive link between NAbs and SpAbs, as naïve IgG NAbs reacting to KLH correlated to IgG levels reacting to whole liver fluke protein extract. Furthermore, in experimentally infected animals, the IgM NAb responses to fluke extract before parasite exposure correlated to the final liver fluke burdens, suggesting that there could be a population of NAbs capable of interacting with fluke molecules and that IgM NAbs could possibility bind and protect the liver fluke’s surface tegument by blocking the onslaught of the host immune system. Further investigation is warranted to determine if bovine NAb levels can be used as a selection marker in breeding programs, either to improve the anti-fluke vaccine efficacy or to improve natural parasite resistance.

## Figures and Tables

**Table 1:** Pearson’s correlation coefficients (*p*-values in parentheses) for the bovine IgG and IgM humoral responses and the liver fluke burdens and wet weights for animals in the vaccinated (*n* = 6) and control (*n* = 6) group of study A. The natural antibody response is assessed to KLH and the specific antibody response to native glutathione *S*-transferase (GST). Significant values (*p* < 0.05) are noted with an asterisk (*).

	IgG								IgM							
	KLH				GST				KLH				GST			
**Day:**	**0**	**42**	**84**	**125**	**0**	**42**	**84**	**125**	**0**	**42**	**84**	**125**	**0**	**42**	**84**	**125**
	**Vaccinated**															
**Fluke Burden**	0.04 (0.94)	−0.44 (0.39)	−0.20 (0.70)	−0.05 (0.93)	0.28 (0.59)	−0.77 (0.07)	−0.78 (0.07)	−0.77 (0.07)	−0.46 (0.36)	−0.18 (0.73)	0.24 (0.64)	−0.41 (0.42)	0.06 (0.91)	−0.75 (0.08)	−0.66 (0.15)	−0.69 (0.13)
**Fluke Weight**	−0.03 (0.95)	−0.35 (0.50)	−0.15 (0.78)	0.06 (0.91)	0.20 (0.70)	−0.83 * (0.04)	−0.82 * (0.04)	−0.81 * (0.04)	−0.60 (0.22)	−0.24 (0.65)	0.10 (0.86)	−0.30 (0.56)	−0.04 (0.94)	−0.85 * (0.03)	−0.75 (0.08)	−0.73 (0.10)
	**Control**															
**Fluke Burden**	−0.29 (0.57)	−0.42 (0.41)	−0.42 (0.41)	−0.39 (0.44)	−0.23 (0.66)	0.56 (0.25)	0.04 (0.94)	−0.55 (0.26)	−0.36 (0.48)	−0.39 (0.46)	−0.76 (0.08)	−0.35 (0.49)	−0.31 (0.55)	0.19 (0.73)	−0.49 (0.32)	−0.37 (0.47)
**Fluke Weight**	−0.19 (0.71)	−0.46 (0.36)	−0.44 (0.38)	−0.12 (0.82)	−0.24 (0.65)	0.29 (0.57)	−0.08 (0.88)	−0.57 (0.24)	−0.28 (0.59)	−0.20 (0.70)	−0.48 (0.33)	−0.20 (0.70)	−0.19 (0.72)	0.45 (0.37)	−0.26 (0.61)	−0.23 (0.66)

**Table 2:** Pearson’s correlation coefficients (*p*-values in parentheses) for the bovine IgG and IgM humoral responses and the liver fluke burdens and wet weights for experimentally infected animals in study A, B and C (*n* = 19). The natural antibody response is assessed to KLH and the specific antibody response to liver fluke’s whole-worm extract (WWE). Significant values (*p* < 0.05) are noted with an asterisk (*). ^†^: datasets that had an outlier removed (*n* = 18).

	IgG				IgM			
	KLH		WWE		KLH		WWE	
	Naïve	Infected	Naïve	Infected	Naïve	Infected ^†^	Naïve ^†^	Infected
**Fluke Burden**	−0.08 (0.74)	0.28 (0.24)	−0.05 (0.85)	−0.19 (0.44)	0.10 (0.69)	0.43 (0.07)	0.56 * (0.02)	0.29 (0.23)
**Fluke Weight**	−0.16 (0.51)	0.21 (0.34)	0.01 (0.97)	−0.09 (0.70)	0.07 (0.77)	0.123 (0.35)	0.25 (0.32)	0.118 (0.44)

## Data Availability

All data are contained within this article or in the supplementary material.

## Supplementary Materials

The following supporting information can be downloaded at: https://www.mdpi.com/article/10.3390/vetsci9020058/s1, Table S1: Each trials study information, including; cattle breed, vaccination, infection and permits, Table S2: Liver fluke burdens and wet weight values for individual animals for all studies, Figure S1: Determination of the optimal assay serum dilution for analysis of NAb levels for IgG (A) and IgM (B) isotypes.

## References

[1] Bruinsma J. (2017). World Agriculture: Towards 2015/2030: An FAO Study.

[2] Woodgate R., Cornell A., Sangster N. (2017). Occurrence, measurement and clinical perspectives of drug resistance in important parasitic helminths of livestock. Antimicrobial Drug Resistance.

[3] Mphahlele M., Molefe N., Tsotetsi-Khambule A., Oriel T. (2019). Anthelmintic Resistance in Livestock. Helminthiasis.

[4] Spithill T.W. (1999). Fasciola gigantica: Epidemiology, control, immunology and molecular biology. Fasciolosis.

[5] Mehmood K., Zhang H., Sabir A.J., Abbas R.Z., Ijaz M., Durrani A.Z., Saleem M.H., Rehman M.U., Iqbal M.K., Wang Y. (2017). A review on epidemiology, global prevalence and economical losses of fasciolosis in ruminants. Microb. Pathog..

[6] Kelley J.M., Elliott T.P., Beddoe T., Anderson G., Skuce P., Spithill T.W. (2016). Current threat of triclabendazole resistance in *Fasciola hepatica*. Trends Parasitol..

[7] Fairweather I., Brennan G., Hanna R., Robinson M., Skuce P. (2020). Drug resistance in liver flukes. Int. J. Parasitol. Drugs Drug Resist..

[8] Cervi L., Rossi G., Masih D. (1999). Potential role for excretory–secretory forms of glutathione-S-transferase (GST) in *Fasciola hepatica*. Parasitology.

[9] Piedrafita D., Estuningsih E., Pleasance J., Prowse R., Raadsma H.W., Meeusen E.N., Spithill T.W. (2007). Peritoneal lavage cells of Indonesian thin-tail sheep mediate antibody-dependent superoxide radical cytotoxicity in vitro against newly excysted juvenile *Fasciola gigantica* but not juvenile *Fasciola hepatica*. Infect. Immun..

[10] Piedrafita D., Parsons J.C., Sandeman R.M., Wood P., Estuningsih S., Partoutomo S., Spithill T.W. (2001). Antibody-dependent cell-mediated cytotoxicity to newly excysted juvenile *Fasciola hepatica* in vitro is mediated by reactive nitrogen intermediates. Parasite Immunol..

[11] Brophy P., Patterson L., Brown A., Pritchard D. (1995). Glutathione S-transferase (GST) expression in the human hookworm Necator americanus: Potential roles for excretory-secretory forms of GST. Acta Trop..

[12] Toet H., Piedrafita D.M., Spithill T.W. (2014). Liver fluke vaccines in ruminants: Strategies, progress and future opportunities. Int. J. Parasitol..

[13] Piedrafita D., Spithill T.W., Dalton J.P., Brindley P.J., Sandeman M.R., Wood P.R., Parsons J.C. (2000). Juvenile *Fasciola hepatica* are resistant to killing In Vitro by free radicals compared with larvae of Schistosoma mansoni. Parasite Immunol..

[14] Morrison C.A., Colin T., Sexton J.L., Bowen F., Wicker J., Friedel T., Spithill T.W. (1996). Protection of cattle against *Fasciola hepatica* infection by vaccination with glutathione S-transferase. Vaccine.

[15] Spithill T.W., Toet H., Rathinasamy V., Zerna G., Swan J., Cameron T., Smooker P.M., Piedrafita D.M., Dempster R., Beddoe T. (2021). 12 Vaccines for Fasciola: New Thinking. Fasciolosis.

[16] Baumgarth N., Tung J.W., Herzenberg L.A. (2005). Inherent specificities in natural antibodies: A key to immune defense against pathogen invasion. Springer Seminars in Immunopathology.

[17] Panda S., Ding J.L. (2015). Natural antibodies bridge innate and adaptive immunity. J. Immunol..

[18] Boes M. (2000). Role of natural and immune IgM antibodies in immune responses. Mol. Immunol..

[19] Holodick N.E., Rodríguez-Zhurbenko N., Hernández A.M. (2017). Defining natural antibodies. Front. Immunol..

[20] Maddur M.S., Lacroix-Desmazes S., Dimitrov J.D., Kazatchkine M.D., Bayry J., Kaveri S.V. (2020). Natural Antibodies: From First-Line Defense Against Pathogens to Perpetual Immune Homeostasis. Clin. Rev. Allergy Immunol..

[21] Hamilton A.M., Lehuen A., Kearney J.F. (1994). Immunofluorescence analysis of B-1 cell ontogeny in the mouse. Int. Immunol..

[22] Hardy R.R., Hayakawa K. (1993). CD5 B cells, a fetal B cell lineage. Advances in Immunology.

[23] Baumgarth N., Herman O.C., Jager G.C., Brown L.E., Herzenberg L.A., Chen J. (2000). B-1 and B-2 cell–derived immunoglobulin M antibodies are nonredundant components of the protective response to influenza virus infection. J. Exp. Med..

[24] Berghof T., Matthijs M., Arts J., Bovenhuis H., Dwars R., Van Der Poel J., Visker M., Parmentier H. (2019). Selective breeding for high natural antibody level increases resistance to avian pathogenic Escherichia coli (APEC) in chickens. Dev. Comp. Immunol..

[25] Sun Y., Parmentier H., Frankena K., Van Der Poel J. (2011). Natural antibody isotypes as predictors of survival in laying hens. Poult. Sci..

[26] Berghof T., Arts J., Bovenhuis H., Lammers A., Van Der Poel J., Parmentier H. (2018). Antigen-dependent effects of divergent selective breeding based on natural antibodies on specific humoral immune responses in chickens. Vaccine.

[27] Thompson-Crispi K., Miglior F., Mallard B. (2013). Genetic parameters for natural antibodies and associations with specific antibody and mastitis in Canadian Holsteins. J. Dairy Sci..

[28] Van Altena S., Peen M., Van der Linden F., Parmentier H., Savelkoul H., Tijhaar E. (2016). Bovine natural antibodies in antibody-dependent bactericidal activity against Escherichia coli and Salmonella Typhimurium and risk of mastitis. Vet. Immunol. Immunopathol..

[29] Ploegaert T., Tijhaar E., Lam T., Taverne-Thiele A., Van der Poel J., Van Arendonk J., Savelkoul H., Parmentier H. (2011). Natural antibodies in bovine milk and blood plasma: Variability among cows, repeatability within cows, and relation between milk and plasma titers. Vet. Immunol. Immunopathol..

[30] De Klerk B., Ducro B., Heuven H., Den Uyl I., Van Arendonk J., Parmentier H., Van der Poel J. (2015). Phenotypic and genetic relationships of bovine natural antibodies binding keyhole limpet hemocyanin in plasma and milk. J. Dairy Sci..

[31] Van Knegsel A., de Vries Reilingh G., Meulenberg S., Van den Brand H., Dijkstra J., Kemp B., Parmentier H. (2007). Natural antibodies related to energy balance in early lactation dairy cows. J. Dairy Sci..

[32] Ochsenbein A.F., Zinkernagel R.M. (2000). Natural antibodies and complement link innate and acquired immunity. Immunol. Today.

[33] Harris J., Markl J. (1999). Keyhole limpet hemocyanin (KLH): A biomedical review. Micron.

[34] Brockwell Y., Spithill T., Anderson G., Grillo V., Sangster N. (2013). Comparative kinetics of serological and coproantigen ELISA and faecal egg count in cattle experimentally infected with *Fasciola hepatica* and following treatment with triclabendazole. Vet. Parasitol..

[35] McCusker P., Toet H., Rathinasamy V., Young N., Beddoe T., Anderson G., Dempster R., McVeigh P., McCammick E., Wells D. (2020). Molecular characterisation and vaccine efficacy of two novel developmentally regulated surface tegument proteins of *Fasciola hepatica*. Vet. Parasitol..

[36] Zerna G., Rathinasamy V.A., Toet H., Anderson G., Dempster R., Spithill T.W., Beddoe T. (2021). Evaluation of Immunogenicity and Efficacy of *Fasciola hepatica* Tetraspanin 2 (TSP2) Fused to E. coli Heat-Labile Enterotoxin B Subunit LTB Adjuvant Following Intranasal Vaccination of Cattle. Vaccines.

[37] Swan J., Sakthivel D., Cameron T.C., Faou P., Downs R., Rajapaksha H., Piedrafita D., Beddoe T. (2019). Proteomic identification of galectin-11 and-14 ligands from *Fasciola hepatica*. Int. J. Parasitol..

[38] Wijffels G.L., Sexton J.L., Salvatore L., Pettitt J.M., Humphris D.C., Panaccio M., Spithill T.W. (1992). Primary sequence heterogeneity and tissue expression of glutathione S-transferases of *Fasciola hepatica*. Exp. Parasitol..

[39] Molina-Hernández V., Mulcahy G., Pérez J., Martínez-Moreno Á., Donnelly S., O’Neill S.M., Dalton J.P., Cwiklinski K. (2015). *Fasciola hepatica* vaccine: We may not be there yet but we’re on the right road. Vet. Parasitol..

[40] Reyneveld G.I., Savelkoul H.F., Parmentier H.K. (2020). Current understanding of natural antibodies and exploring the possibilities of modulation using veterinary models. A review. Front. Immunol..

[41] de Klerk B., Emam M., Thompson-Crispi K.A., Sargolzaei M., van der Poel J.J., Mallard B.A. (2018). A genome-wide association study for natural antibodies measured in blood of Canadian Holstein cows. BMC Genom..

[42] Ploegaert T., Wijga S., Tijhaar E., Van Der Poel J., Lam T., Savelkoul H., Parmentier H., Van Arendonk J. (2010). Genetic variation of natural antibodies in milk of Dutch Holstein-Friesian cows. J. Dairy Sci..

[43] Wijga S., Bovenhuis H., Bastiaansen J., Van Arendonk J., Ploegaert T., Tijhaar E., Van Der Poel J. (2013). Genetic parameters for natural antibody isotype titers in milk of Dutch Holstein-Friesians. Anim. Genet..

[44] Wijga S., Parmentier H., Nieuwland M., Bovenhuis H. (2009). Genetic parameters for levels of natural antibodies in chicken lines divergently selected for specific antibody response. Poult. Sci..

[45] Parmentier H.K., Lammers A., Hoekman J.J., Reilingh G.D.V., Zaanen I.T., Savelkoul H.F. (2004). Different levels of natural antibodies in chickens divergently selected for specific antibody responses. Dev. Comp. Immunol..

[46] Prada Jiménez de Cisneros J., Stear M.J., Mair C., Singleton D., Stefan T., Stear A., Marion G., Matthews L. (2014). An explicit immunogenetic model of gastrointestinal nematode infection in sheep. J. R. Soc. Interface.

[47] Stills H.F. (2005). Adjuvants and antibody production: Dispelling the myths associated with Freund’s complete and other adjuvants. ILAR J..

[48] Dalton J.P., Robinson M.W., Mulcahy G., O’Neill S.M., Donnelly S. (2013). Immunomodulatory molecules of *Fasciola hepatica*: Candidates for both vaccine and immunotherapeutic development. Vet. Parasitol..

[49] Spickler A.R., Roth J.A. (2003). Adjuvants in veterinary vaccines: Modes of action and adverse effects. J. Vet. Intern..

[50] Cecchini S., Rufrano D., Caputo A. (2019). Natural antibodies and their relationship with total immunoglobulins and acquired antibody response in goat kid (*Capra hircus*, L. 1758) serum. Vet. Immunol. Immunopathol..

[51] Sandmeier F.C., Tracy C.R., Dupré S., Hunter K. (2012). A trade-off between natural and acquired antibody production in a reptile: Implications for long-term resistance to disease. Biol. Open.

[52] Seebach J.D., Yamada K., McMorrow I.M., Sachs D.H., DerSimonian H. (1996). Xenogeneic human anti-pig cytotoxicity mediated by activated natural killer cells. Xenotransplantation.

[53] Sulaiman A.A., Zolnierczyk K., Japa O., Owen J.P., Maddison B.C., Emes R.D., Hodgkinson J.E., Gough K.C., Flynn R.J. (2016). A trematode parasite derived growth factor binds and exerts influences on host immune functions via host cytokine receptor complexes. PLoS Path..

[54] Piedrafita D., Raadsma H., Prowse R., Spithill T.W. (2004). Immunology of the host–parasite relationship in fasciolosis (*Fasciola hepatica* and *Fasciola gigantica*). Can. J. Zool..

[55] Ravidà A., Aldridge A.M., Driessen N.N., Heus F.A., Hokke C.H., O’Neill S.M. (2016). *Fasciola hepatica* surface coat glycoproteins contain mannosylated and phosphorylated *N*-glycans and exhibit immune modulatory properties independent of the mannose receptor. PLoS Negl. Trop. Dis..

[56] Ravidà A., Cwiklinski K., Aldridge A.M., Clarke P., Thompson R., Gerlach J.Q., Kilcoyne M., Hokke C.H., Dalton J.P., O’Neill S.M. (2016). *Fasciola hepatica* surface tegument: Glycoproteins at the interface of parasite and host. Mol. Cell Proteom..

[57] New J.S., King R.G., Kearney J.F. (2020). Glycan Reactive Natural Antibodies and Viral Immunity. Viral Immunol..

[58] Chauvin A., Boulard C. (1996). Local immune response to experimental *Fasciola hepatica* infection in sheep. Parasite.

[59] Frigerio S., Da Costa V., Costa M., Festari M.F., Landeira M., Rodríguez-Zraquia S.A., Härtel S., Toledo J., Freire T. (2020). Eosinophils Control Liver Damage by Modulating Immune Responses against *Fasciola hepatica*. Front. Immunol..

