# Supplementary material for: Bovine Natural Antibody Relationships to Specific Antibodies and Fasciola hepatica Burdens after Experimental Infection and Vaccination with Glutathione S-Transferase
Source: Vet Sci. 2022 Jan 31;9(2):58. doi: 10.3390/vetsci9020058 (PMC8876122; doi:10.3390/vetsci9020058)
Supplement: Supplementary file 1 [file vetsci-09-00058-s001.zip › vetsci-1536284-supplementary.pdf]

Supplementary Materials

Table S1. Each trials study information, including; cattle breed, vaccination, infection and permits.

Study	Animal breed	Control group formulation	Vaccination days	Infective dose	Infection /Sacrifice day	Study #	Ethics permit	AVPMA	Dept Agriculture permit
A	Angus/ Angus Hereford cross	PBS in FCA/FIA	0, 28	350	42/ 125-126	AVBAB17039	UNE: AEC17/084	PER7250	2018/053
B	Angus	PBS only	0, 33	520	61/ 145-146	VAVB3035	UNE: AEC14/043	PER7250	N/A[1]
C	Angus	PBS in FCA/FIA	0, 30	535	44/ 126-128	VAVB3150	CSIRO: AEC15/16	PER7250	2015/093

[1] Department of Agriculture Permit not applicable. Animal used in study B were control group animals that only received saline in the vaccine formulation. Abbreviations: PBS: Phosphate buffered saline, FCA: Freund's Complete Adjuvant, FIA: Freund's Incomplete Adjuvant, met: metacercariae.

Table S2. Liver fluke burdens and wet weight values for individual animals for all studies.

Group	Treatment	Challenge met no.	Animal ID	Number of flukes	Fluke wet weight (g)
Vaccinated (Study A)	Native GST + tegumental antigen (200 µg each) + FCA/FIA	350	V1	105	3.6
			V2	36	1.4
			V3	61	2.1
			V4	28	1.3
			V5	23	0.5
			V6	35	1.2
			Average	48 ± 30.8	1.7 ± 1.1
Control (Study A)/ Ex. Infected	PBS + FCA/FIA	350	C1	66	2.0
			C2	77	3.9
			C3	111	4.4
			C4	48	1.2
			C5	68	2.0
			C6	63	1.8
			Average	72 ± 21.2	2.6 ± 1.3
Ex. Infected (Study B)	PBS	520	C7	113	5.8
			C8	87	3.6
			C9	84	1.8

			C10	160	6.6
			C11	73	4.4
			C12	151	6.7
			Average	111 ± 36.8	4.8 ± 1.9
Ex. Infected (Study C)	PBS + FCA/FIA	535	C13	159	14.7
			C14	82	7.0
			C15	89	8.5
			C16	87	5.5
			C17	130	10.8
			C18	84	4.2
			C19	122	6.9
			Average	108 ± 29.8	8.2 ± 3.5
			Ex. Infected Average	98 ± 33.4	5.35 ± 3.4

Abbreviations: Met: Metacercaria,

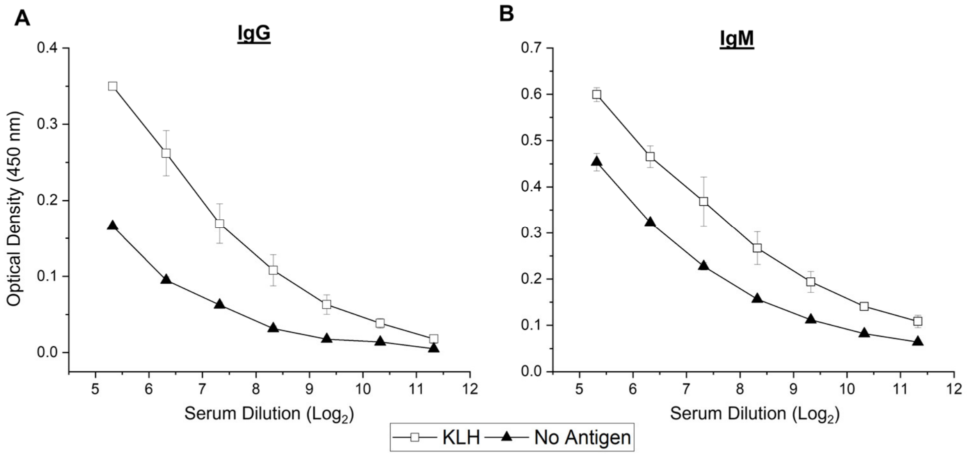

Figure S1. Determination of the optimal assay serum dilution for analysis of NAb levels for IgG (A) and IgM (B) isotypes. Displayed are the pooled (n= 12, study A) naïve bovine serum NAb responses to KLH (empty squares) and to no antigen coated wells (filled triangles). Results are displayed as the mean optical density (450 nm) of duplicates with error bars representing the standard deviation of the mean.
